# Comparing Discrete Choice Experiment with Swing Weighting to Estimate Attribute Relative Importance: A Case Study in Lung Cancer Patient Preferences

**DOI:** 10.1177/0272989X231222421

**Published:** 2024-01-04

**Authors:** J. Veldwijk, I. P. Smith, S. Oliveri, S. Petrocchi, M. Y. Smith, L. Lanzoni, R. Janssens, I. Huys, G. A. de Wit, C. G. M Groothuis-Oudshoorn

**Affiliations:** Erasmus School of Health Policy & Management, Erasmus University, Rotterdam, the Netherlands; Erasmus Choice Modelling Centre, Erasmus University, Rotterdam, the Netherlands; Julius Center for Health Sciences and Primary Care, University Medical Center Utrecht, Utrecht University, Julius Centrum, Utrecht, the Netherlands; Julius Center for Health Sciences and Primary Care, University Medical Center Utrecht, Utrecht University, Julius Centrum, Utrecht, the Netherlands; Applied Research Division for Cognitive and Psychological Science, IEO, European Institute of Oncology IRCCS, Milan, Italy; Applied Research Division for Cognitive and Psychological Science, IEO, European Institute of Oncology IRCCS, Milan, Italy; Alexion AstraZeneca Rare Disease, Boston, MA, USA; Department of Regulatory and Quality Sciences, School of Pharmacy, University of Southern California, Los Angeles, CA, USA; Applied Research Division for Cognitive and Psychological Science, IEO, European Institute of Oncology IRCCS, Milan, Italy; Department of Pharmaceutical and Pharmacological Sciences, KU Leuven, Leuven, Belgium; Department of Pharmaceutical and Pharmacological Sciences, KU Leuven, Leuven, Belgium; Julius Center for Health Sciences and Primary Care, University Medical Center Utrecht, Utrecht University, Julius Centrum, Utrecht, the Netherlands; Department of Health Sciences, Faculty of Science, Vrije Universiteit Amsterdam & Amsterdam Public Health Research Institute, Amsterdam, the Netherlands; Health Technology and Services Research (HTSR), Faculty of Behavioural Management and Social Sciences, University of Twente, Enschede, the Netherlands

**Keywords:** discrete choice experiment, swing weighting, attribute relative importance, patient preferences, lung cancer treatment

## Abstract

**Introduction:**

Discrete choice experiments (DCE) are commonly used to elicit patient preferences and to determine the relative importance of attributes but can be complex and costly to administer. Simpler methods that measure relative importance exist, such as swing weighting with direct rating (SW-DR), but there is little empirical evidence comparing the two. This study aimed to directly compare attribute relative importance rankings and weights elicited using a DCE and SW-DR.

**Methods:**

A total of 307 patients with non–small-cell lung cancer in Italy and Belgium completed an online survey assessing preferences for cancer treatment using DCE and SW-DR. The relative importance of the attributes was determined using a random parameter logit model for the DCE and rank order centroid method (ROC) for SW-DR. Differences in relative importance ranking and weights between the methods were assessed using Cohen’s weighted kappa and Dirichlet regression. Feedback on ease of understanding and answering the 2 tasks was also collected.

**Results:**

Most respondents (>65%) found both tasks (very) easy to understand and answer. The same attribute, survival, was ranked most important irrespective of the methods applied. The overall ranking of the attributes on an aggregate level differed significantly between DCE and SW-ROC (*P* < 0.01). Greater differences in attribute weights between attributes were reported in DCE compared with SW-DR (*P* < 0.01). Agreement between the individual-level attribute ranking across methods was moderate (weighted Kappa 0.53–0.55).

**Conclusion:**

Significant differences in attribute importance between DCE and SW-DR were found. Respondents reported both methods being relatively easy to understand and answer. Further studies confirming these findings are warranted. Such studies will help to provide accurate guidance for methods selection when studying relative attribute importance across a wide array of preference-relevant decisions.

**Highlights:**

- Both DCEs and SW tasks can be used to determine attribute relative importance rankings and weights; however, little evidence exists empirically comparing these methods in terms of outcomes or respondent usability.
- Most respondents found the DCE and SW tasks very easy or easy to understand and answer.
- A direct comparison of DCE and SW found significant differences in attribute importance rankings and weights as well as a greater spread in the DCE-derived attribute relative importance weights.

As health care systems evolve toward more patient-centered care, there has been an increased interest in using patient preferences to support decision making to optimize care from product and process development to authorization and reimbursement.^1–3^ Patient preference assessments measure what patients value in their health care and can be used to compare different aspects of care and tradeoffs patients find acceptable.^4,5^ Patient preferences can be elicited using a variety of methods.^6,7^

One frequently used method to elicit and quantify patient preferences is a discrete choice experiment (DCE).^6^ DCEs are based on the random utility theory and require respondents to answer several choice tasks in which they are presented with multiple alternatives representing different health care options. The alternatives are described using a set of attributes with varying levels.^4,8,9^ From these alternatives, respondents choose the option with the highest personal utility.^9–12^ Based on the choices respondents make, the impact each attribute has on the utility is estimated, and the relative importance of the included attributes can be inferred from these estimates.^9,13,14^ DCEs can thus be used to prioritize attributes of different care paths, calculate the relative importance of these attributes, and identify potential tradeoffs that patients are willing to make between these different attributes. The validity of DCE findings is well supported.^4,15^ However, DCEs have been criticized for being complex for both researchers and respondents. First, DCEs require expert knowledge for generating formal experimental designs^16^ and running the required complex statistical modelling techniques.^9^ Second, DCEs are generally considered to be cognitively burdensome to respondents, making them less than ideal for participants who have cognitive impairments.^17,18^ In addition, they require relatively large sample sizes,^19^ making them inappropriate for administration in, for example, rare disease populations. The combination of expert input and large sample size renders relatively high study costs and long study duration.^18,19^

Researchers as well as stakeholders who use preference information (i.e., representatives from the pharmaceutical industry and regulatory and reimbursement bodies) have expressed the need to compare DCEs to other, simpler methods.^22^ This will help guide method selection for use in patient preference studies that are budget and or time sensitive, conducted in rare disease areas, and for which Marginal Rate of Substitution (MRS) or predicted uptake are not among the required outcome measures (e.g., prioritization of unmet medical needs or endpoint selection for clinical trials).

Swing weighting (SW) has been identified as a “simpler” preference elicitation method and was identified by researchers and other stakeholders as a promising method^23^ to be applied when attribute importance is an outcome of interest to inform decision making.^24^ In SW tasks, respondents are presented with a list of attributes used to define a health care treatment option. Each attribute on the list shows the “swing” from the attribute’s worst level to its best level (worst and best levels are determined a priori). The participant ranks these swings based on how important improving that attribute is to them. SW tasks are followed by a point allocation (PA) or direct rating (DR) task. In such a task, respondents state the value of each swing either by allocating a fixed number of points (usually out of 100 points) between the “swings” or by directly rating each swing on a standard point scale, with the top-ranked swing automatically receiving the maximum possible number of points (usually 100 points).^25^ The results of an SW task can then be used to identify attribute priorities, and the relative importance weights of each ranked swing can be calculated using the proportion of points given to each swing.^26,27^ This type of rating scale is an often-used way to measure the relative importance, and thus utility, of attributes. While some consider SW as simply a ranking method,^27^ others argue that given the application of multiattribute value functions, SW (like DCE) is based on the concepts and axioms described by von Neumann and Morgenstern^28^ and is embedded in multiattribute utility theory.^29,30^ The key difference is that SW does not include a “random” component as choices in SW are deterministic in nature.^31^ This enables researchers to directly capture relative attribute weights at an individual level (whereas for a DCE, the relative importance weights are estimated as a secondary outcome available only after applying econometric modeling) and can be done with smaller sample sizes and a greater number of attributes compared with DCE studies.^26,31^ SW also does not require a formal experimental design, making them easier to develop, and they are believed to be cognitively easier to complete than a DCE task.^26,31^

While both DCE and SW have been implemented in health care preference research, empirical evidence directly comparing DCE and SW outcomes in terms of attribute relative importance and ease of comprehension and completion is largely lacking.^31,32^ Where some studies compared DCE to other methods in different clinical settings (e.g., DCE versus ordered categorical,^33^ DCE versus best-worst-scaling,^34–36^ or DCE versus thresholding,^37,38^ this study aimed to address this gap in knowledge by empirically comparing DCE and swing weighting with direct rating (SW-DR)–derived attribute relative importance rankings and weights. Since both methods claim they can be used to determine attribute relative importance rankings and weights, applying them to a similar research question should result in comparable estimates.

## Methods

### Study Context and Ethics

The outcomes of a study assessing the preferences of Italian and Belgian patients with non–small-cell lung cancer (NSCLC) for treatment was used for this comparative analysis. Details on the study design have been published elsewhere.^39,40^ This case study was identified as suitable for the comparison of DCE and SW-DR due to the potentially fragile physical state or diminished cognitive status of the patients.^41–44^ The current study included DCE and SW exercises in the ways these methods typically would be applied to answer a particular clinical research question. The study was approved by the Ethical Committee of the European Institute of Oncology IRCCS (IEO, Milan, Italy; reference R1142/20-IEO 1206) and the Ethische Commissie Onderzoek UZ/KU Leuven (Belgium; reference S63007).

### Respondents and Recruitment

Patients with NSCLC were recruited through clinical partners in Italy and Belgium. Respondents were selected and referred to the PREFER research team by the treating oncologists at cancer treatment centers in Belgium and in Italy.^40^ To be eligible, patients had to understand Italian or Dutch, be 18 y or older, and have a histologic or cytologic diagnosis of NSCLC as evaluated by clinicians. Patients were not eligible if they (as evaluated by the clinician): 1) had cognitive impairments rendering the participant incapable of informed consent or 2) were unable to understand the study materials.

### Attribute and Level Selection

Attributes and levels were identified and refined according to best practices and guidelines.^9,45–47^ This included a literature review, 6 nominal group technique–based focus groups in Italy and Belgium with NSCLC patients,^48,49^ and a multistakeholder discussion with clinicians and preference experts.^50^ Five attributes with 3 levels each were identified as relevant for the study (see Table 1).

**Table 1:** Attributes and Levels Included in the Discrete Choice Experiment and the Swings Used in the Swing Weighting

Attribute		Level
How the treatment is being given to you (mode)		Oral treatment Intravenous infusion lasting 24 h Intravenous infusion lasting 12 h
Chance of surviving 5 y after beginning cancer treatment (5-y survival)		10% 20% 40%
Risk of persistent skin problems (skin problems)		10% 20% 40%
Risk of being extremely tired (tiredness)		10% 40% 60%
Severity of hair loss (hair)		No hair loss Weakening/thinning of the hair Complete loss of hair
Swing	Worst	Best
How the treatment is being given to you	Intravenous infusion lasting 24 h	Oral treatment
Chance of surviving 5 y after beginning of the cancer treatment	10%	40%
Risk of persistent skin problems	40%	10%
Risk of being extremely tired	60%	10%
Severity of hair loss	Complete loss of hair	No hair loss

### DCE Experimental Design

**Figure 1:**
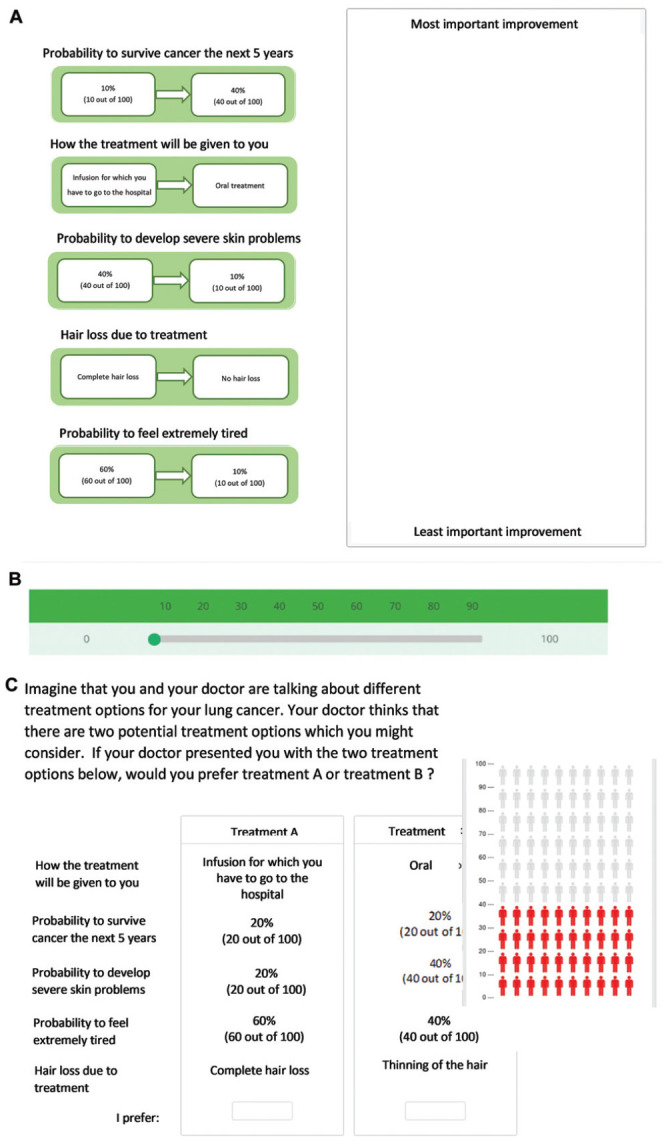
Illustration of survey elements. (A) Swing weighting (SW) ranking task in which respondents sort the swings in attributes from worst level to best level by priority for improvement in a treatment. (B) SW direct rating task in which patients rate the swings relative on a scale from 0 (not at all important) to 100 (as important as the most important improvement). (C) Discrete choice experiment choice task in which respondents choose their most preferred treatment (pop-up shown to explain risk attribute).

A Bayesian D-efficient design consisting of 2-unlabeled alternative-forced-choice tasks was constructed for the DCE using Ngene (ChoiceMetrics, Sydney, Australia).^16,51^ A total of 36 unique choice tasks were generated, which were divided over three 12-choice task blocks. Respondents were randomly assigned to complete 1 of those blocks. Attribute prior information for DCE design optimization was generated using previously published literature and best guesses. The survey was pilot tested among respondents in Italy (*N* = 50), with the outcomes of a conditional logit model used to inform the final experimental design. Interactions between the attributes “5-y survival” and, respectively, “Risk of long-lasting skin problems,”“Risk of extreme tiredness,” and “Mode of administration” were accounted for in this design. An example of a DCE choice task can be found in Figure 1.

### SW Design

An SW-DR task was developed using the same attributes and levels used in the DCE. In the SW section, respondents were first asked to choose the attribute they preferred to swing from the lowest (worst) to the highest (best) level. Respondents were asked to rank all other swings subsequently from most to least preferred (see Figure 1A). The order in which the swings were presented was randomized in this section. In the DR section, respondents were asked to rate each of the swings relative to the others by giving it between 0 and 100 points, except for the highest ranked swing, which automatically received 100 points^25^ (see Figure 1B). This reflects the relative valuation of the importance of the different swings. Respondents were instructed on what this relative rating means as follows: “If you give 50 points to improve a feature, it means that you think improving it is half as important as improving the top ranked attribute because you gave it half as many points.” This unrestricted valuation is assumed to be simpler for respondents than PA from a fixed pool and has been found to be more reliable than restricted PA methods,^52–55^ making it more suitable for this study population, who may have more fragile physical states or diminished cognitive status.^41–44^

### Survey

Both the DCE and SW-DR tasks were included as parts of a one-time online survey with respondents able to pause and return to the survey. The survey was programmed in Sawtooth software (lighthouse studio 9.13) and consisted of 6 parts. First, respondents were informed about the study and provided consent for data collection prior to answering sociodemographic and medical history–related questions. Second, respondents watched 2 different educational videos consisting of text and animations with voiceovers giving 1) an introduction with information on lung cancer and detailed descriptions of the attributes and levels included and (2) instructions on how to complete the first-choice task. Third, respondents were randomly assigned to receive either the DCE or SW task first to avoid any ordering effects. Fourth, respondents completed quality of life–related questions (EQ-5D).^56,57^ Fifth, respondents watched a video with instructions on how to complete the second choice task. Finally, respondents were asked to complete psychosocial measures including measures of health literacy.^58,59^

After each choice task, respondents were given 2 feedback questions about ease of understanding and answering the choice tasks on a 5-point Likert-type scale ranging from very easy to very difficult. The survey was pretested with 5 lung cancers patients in think-aloud interviews.

### Statistical Analysis

Contrary to common practice in applied preference research, in this study, only surveys of respondents who completed both DCE and SW choice tasks were included in the analysis to facilitate within-person comparisons. One respondent was excluded from the data set due to flatlining behavior (defined as always choosing A or always choosing B). Statistical analysis was performed with Nlogit version 6 and R version 4.0.4. A significance level of *P* < 0.05 was used for all analyses. All analyses were performed separately for data from Italy and Belgium to ensure most accurate methods comparison measures and avoid conflating potential scale heterogeneity between countries.^60^

#### Respondent background characteristics

Respondent background characteristics (including general demographic and medical history information) were categorized and are presented as counts with percentages.

#### DCE analysis

Random parameter logit models (RPLs) were used to analyze the DCE data. Such models adjust for the fact that panel data were collected and adjusted for the multilevel structure of the data.^9,13^ In addition, these models allow to include attribute (levels) as random parameters to adjust for the effect of preference heterogeneity.^9,13^ All risk and benefit attributes were assumed to be linear, and the categorical attributes were dummy coded. The significance level of the standard deviation of the attributes was used to test which attributes should be included in the final model as random parameters (assuming normal distributions) to account for preference heterogeneity. The utility equation below formulates the outcomes of these procedures and displays the final utility model tested in the analysis. The systematic utility component (V) describes the measurable utility of a specific treatment based on the attributes included in the DCE. The β_1_–β_7_ coefficients represent the attribute-level estimates indicating the relative importance of each attribute level for individual *i*. A constant term was included in model exploration (i.e., to test for reading order bias), but it was found to be insignificant and removed from the final model.

$$\begin{array}{l} V=\beta_{1,i}*{\mathrm{Mode}}_{\mathrm{infusion}\;\mathrm{at}\;\mathrm{hospital}\;\mathrm{for}\;\mathrm{12}\;\mathrm{hours}}\\ +\beta_{2,i}*{\mathrm{Mode}}_{\mathrm{infusion}\;\mathrm{at}\;\mathrm{hospital}\;\mathrm{for}\;\mathrm{24}\;\mathrm{hours}}\\ +\beta_{3,i}*5-\mathrm{year}\;\mathrm{Survival}\\ +\beta_{4,i}*\mathrm{Risk}\;\mathrm{of}\;\mathrm{long}-\mathrm{lasting}\;\mathrm{skin}\;\mathrm{problems}\\ +\beta_{5,i}*\mathrm{Risk}\;\mathrm{of}\;\mathrm{extreme}\;\mathrm{tiredness}\\ +\beta_{6,i}\mathrm{Hair}\;{\mathrm{loss}}_{\mathrm{some}\;\mathrm{loss}}+\beta_{7,i}\mathrm{Hair}\;{\mathrm{loss}}_{\mathrm{no}\;\mathrm{loss}} \end{array}$$

A choice task-order variable was included in the model as an interaction term with the attribute levels to test whether the task order (i.e., DCE first or SW first) influenced the outcomes, which turned out insignificant. Prespecified interaction terms that significantly contributed to model fit (as assessed using a log-likelihood [LL] ratio test) were included in the model. Individual specific conditional parameter estimates were estimated for each respondent using the final model. Individual attribute weights and rankings were calculated with these parameter estimates (by calculating the total impact of each attribute on utility and standardizing to a total of 100, where the highest weight represents highest rank) and averaged to estimate the mean population weights and rankings.

#### SW analysis

The SW analysis was performed by analyzing the patients’ rankings of the attributes and the points allocated to the different attributes. The individual attribute relative importance weights were calculated using both the rank-ordered centroid (ROC) weight method and the DR weight method per patient. The ROC weight method calculates a relative weight representing the distance between adjacent ranks on an ordinal or normalized scale.^61^

The ROC weight for an attribute with rank *i* equals (in case of 5 attributes):

$$w_{i}=\;\frac{1}{5}\;\sum_{n=i}^{5}\frac{1}{n},\;i=1,\;\ldots ,5.$$

The DR method is used to generate individual proportional weights for an attribute with rank *i* and allocated points *p_i_* and equals (in case of 5 attributes):

$$w_{i}=\frac{p_{i}}{\sum_{i=1}^{5}p_{i}},\;i=1,\ldots ,5.$$

These individual weights were averaged over all patients per country to obtain the average weights, which are the equivalent of the attribute relative importance weights resulting from DCEs.

#### Comparison between methods

##### Respondent feedback

Frequencies and chi-square tests were conducted to compare the feedback of respondents regarding their perceived difficulty in understanding and answering the DCE and SW questions.

##### Comparing attribute importance ranking

Based on the outcomes of the RPL of the DCE and the SW-ROC, attribute ranking was compared. Ranking agreement (based on individual-level estimates from the DCE and SW-ROC) was evaluated with Cohen’s weighted kappa, which measures interrater reliability while accounting for chance similarities in rating.^62,63^ Differences in the ranking based on DCE and SW-ROC were analyzed and tested with an ordered logit model.^64^

##### Comparing attribute importance weighting

Based on the outcomes of the RPL of the DCE and the SW-DR, attribute weighting was compared. Differences in the weighting based on DCE and SW-DR were analyzed and tested using Dirichlet regression models.^65^ Dirichlet regression models can be regarded as a generalization of beta regression models for proportions and percentages and are particularly suited for the analysis of compositional data (i.e., for weights that add up to 1).^66^ In a Dirichlet regression model, the aggregate attribute weights are assumed to be distributed with a Dirichlet distribution with parameters *µ_i_, i* = 1,…, 5, mean attribute weights that add up to 1, and a precision parameter *ϕ* (according to the so-called alternative parametrization).^67^ The mean attribute weights are modeled with a logit link function similar to logistic regression:

$$\mathrm{logit}\left(\mu_{i}\right)=\eta_{i}=\beta_{0,i}+\beta_{1,i}D_{\mathrm{SW}},\;i=1,\ldots ,5$$

Here, the logit of μ for individual i is equal to the linear predictor η and is modeled with an intercept $\beta_{0,i}$ , representing the DCE, and with a dummy variable D_SW_ for the method as covariate. We defined the attribute 5-y survival as the base category, with $\beta_{0,\mathrm{survival}}=\beta_{1,\;\mathrm{survival}\;}=0$ . In this way, the corresponding values of *µ_i_* equal

$$\mu_{i}=\frac{e^{\eta_{i}}}{\sum_{j=1}^{5}e^{\eta_{i}}}\;\mathrm{and}\;\mu_{\mathrm{survival}}=\frac{1}{\sum_{j=1}^{5}e^{\eta_{i}}}.$$

The precision parameter is modelled with a log link function with method as covariate:

$$\log\left(\varphi \right)=\alpha_{0}+\alpha_{1}D_{\mathrm{SW}}.$$

The parameter estimates β_1,i_ can be interpreted as odds ratios after exponentiation relative to survival as base category.^66^ Maximum likelihood estimation is used for obtaining the parameter estimates.^68^ Finally, covariates were added to the models to correct for possible effects of method, for educational level, health literacy, gender, age, cancer stage, and treatment history.

## Results

### Demographics

A sample of *n* = 307 NSCLC patients was obtained from *N* = 560 requests to patients (*n* = 159 declined invite; *n* = 94 withdrew consent). No significant differences were found between the countries in respondents’ gender, age, cancer stage, or family history of cancer. Respondents in both countries differed significantly in family and relationship status, χ^2^(3) = 8.1, *P* = 0.045; education level, χ^2^(2) = 7.248, P = 0.027; and health literacy, *t* (305) = −6.591, *P* < 0.001. Patient demographic information can be found in Table 2.

**Table 2:** Demographic Characteristics of the Sample

		Italy (*n* = 158)		Belgium (*n* = 149)	
		*n*	%	*n*	%
Sex	Male	88	55.7	89	59.7
Age at survey completion, y	≥71	56	35.4	45	30.2
Education	No degree	0	0	6	4
	Primary school	12	7.6	6	4
	Middle school	37	23.4	30	20.1
	Secondary school	51	32.3	51	34.2
	Professional degree	19	12	26	17.4
	Bachelor’s degree	4	2.5	0	0
	Master’s degree	26	16.5	14	9.4
	Postgraduate degree	5	3.2	2	1.3
	Other	4	2.5	14	9.4
Family and relationship status	Single no children	15	9.5	18	12.1
	Single with children	12	7.6	17	11.4
	Partner with children	64	40.5	38	25.5
	Partner no children	67	42.4	76	51
Family history of lung cancer	Yes	45	28.5	37	24.8
Cancer stage	I, II	78	49.4	65	43.6
	III, IV	80	50.6	84	56.4
Type of treatment	No treatments	21	13.3	0	0
	Surgery	94	59.5	78	52.3
	Chemotherapy	55	34.8	88	59.1
	Immunotherapy	35	22.2	78	52.3
	Radiotherapy	35	22.2	46	30.9
	Other	18	11.4	12	8.1
	Don’t know	3	1.9	0	0
Lines of treatment	No treatment	72	45.6	72	48.3
	1 treatment	34	21.5	14	9.4
	2 treatments	14	8.9	15	10.1
	3 treatments	17	10.8	48	32.2
	>3 treatments	21	13.3	0	0
Age when diagnosed, y	<55	28	17.7	21	14.1
	55–64	48	30.4	57	38.2
	65–74	57	36.1	59	39.6
	≥75	25	15.8	12	8.1
Health literacy (newest vital sign)	Very limited literacy	7	4.4	12	8.1
	Limited literacy	40	25.3	32	21.5
	Adequate literacy	111	70.3	105	70.5

### Respondent Feedback

Most respondents found the DCE and SW tasks very easy or easy to understand and answer (74.6% and 64.5% for DCE and 73.0% and 69.7% for SW, respectively, in Italy and Belgium). The ease of understanding and answering the DCE and understanding the SW task was associated with educational level, with those who had higher levels of education reporting greater ease (*P* < 0.001).

### Comparing Attribute Importance Ranking

Table 3 shows attribute ranks for the 2 methods separately per country (Appendix Tables A1 and A2 show the original attribute-level estimates of the DCE and the ROC and DR estimates of the SW that were used for these calculations). Five-year survival was the most important attribute for most of the respondents, irrespective of the method. Agreement between the ranking of the DCE and SW-ROC was moderate with weighted Kappa correlation coefficients varying between 0.53 and 0.55. Despite the similar ranking of the 5-y survival and tiredness attributes, the overall ranking of the attributes differed significantly between DCE and SW-ROC tasks for both countries (χ^2^ = 2042.9, 4 df, *P* < .0001 for Italy; χ^2^ = 1932.5, 4 df, *P* < .0001 for Belgium; Table 4). For the Italian respondents, the attributes of mode and hair swapped their rank (third or fifth) depending on the method. For the Belgian respondents, the attributes of mode, skin problems, and hair changed ranking between being third, fourth, or fifth most important.

**Table 3:** Attribute Rank and Weight (95% Confidence Interval) Based on DCE and SW-DR and Rank and Weight (*s*) for SW-ROC Separately for Italy and Belgium

	Italy						Belgium					
	DCE		SW-DR		SW-ROC		DCE		SW-DR		SW-ROC	
	Rank	Weight (95% CI)	Rank	Weight (95% CI)	Rank	Weight (*s*)	Rank	Weight (95% CI)	Rank	Weight (95% CI)	Rank	Weight (*s*)
Mode of administration	5	0.05 (0.04–0.06)	3	0.18 (0.15 – 0.19)	3	0.16 (0.12)	5	0.02 (0.02–0.02)	4	0.16 (0.14–0.17)	3	0.14 (0.12)
5-y survival	1	0.63 (0.61–0.66)	1	0.33 (0.31–0.34)	1	0.43 (0.08)	1	0.59 (0.57–0.62)	1	0.31 (0.30–0.33)	1	0.42 (0.10)
Risk of long-lasting skin problems	4	0.08 (0.07–0.08)	4	0.16 (0.15–0.17)	4	0.14 (0.07)	4	0.08 (0.08–0.09)	3	0.18 (0.17–0.19)	4	0.14 (0.06)
Risk of extreme tiredness	2	0.16 (0.14–0.17)	2	0.19 (0.18–0.20)	2	0.18 (0.09)	2	0.20 (0.18–0.22)	2	0.22 (0.21–0.23)	2	0.20 (0.08)
Hair loss	3	0.08 (0.08–0.09)	5	0.14 (0.13–0.15)	5	0.10 (0.08)	3	0.10 (0.09–0.11)	5	0.13 (0.12–0.14)	5	0.11 (0.10)

CI, confidence interval; DCE, discrete choice experiement; *s*, standard deviation; SW-DR, swing weighting with direct rating; SW-ROC, swing weighting with rank order centroid method.

**Table 4:** Rank-Ordered Logit Model^a^ Beta Parameters (Mean and SE) Comparing SW-ROC and DCE Including Likelihood Ratio Test and Dirichlet Regression Odds Ratios (SW-DR Compared with DCE and 95% CI) Including the Dispersion Parameter (Ln Phi)

		5-y Survival	Mode of Administration	Risk of Long-Lasting Skin Problems	Risk of Extreme Tiredness	Hair Loss	Ln Phi
Rank-ordered logit							—
Italy	Mean	0.09	2.17*	1.12*	−0.14	Ref	—
	(SE)	(0.37)	(0.23)	(0.21)	(0.22)		—
Likelihood ratio test, χ^2^ = 2,042.9(df = 4), *P* < 0.001							
Belgium	Mean	0.62*	3.58*	1.48*	0.70*	Ref	—
	(SE)	(0.31)	(0.35)	(0.22)	(0.23)		—
Likelihood ratio test, χ^2^ = 1,932.5(df = 4), *P* < 0.001							
Dirichlet regression							
Italy	OR	Ref	4.87	3.80	2.46	2.58	0.75
	(95% CI)		(4.13, 5.74)	(3.24, 4.45)	(2.14, 2.83)	(2.22, 3.03)	(0.64, 0.87)
Belgium	OR	Ref	6.41	3.41	2.15	1.67	1.02
	(95% CI)		(5.27, 7.80)	(2.90, 4.01)	(1.87, 2.48)	(1.42, 1.98)	(0.87, 1.20)

a The coefficients represent the effect of the method used to elicit the rank on the relative importance of the treatment attributes relative to hair loss; a positive coefficient represents increasing importance, while a negative coefficient represents decreasing importance. CI, confidence interval; DCE, discrete choice experiement; OR, odds ratio; SE, standard error; SW-DR, swing weighting with direct rating; SW-ROC, swing weighting with rank order centroid method.

* *P* < 0.01.

### Comparing Attribute Importance Weighting

**Figure 2:**
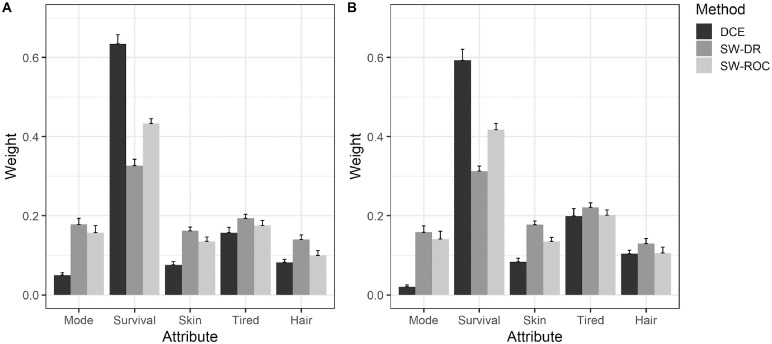
Relative attribute ranking and weights for Belgium (A) and Italy (B) calculated based on the discrete choice experiment (DCE) data and the swing weighting (SW) data, using both direct ranking (DR) and rank-ordered centroid (ROC).

The weights of all the attributes differed substantially between DCE and SW-DR (Table 3 and Figure 2). The largest difference was found for the weight of “5-y survival,” which was much greater for the DCE (59%–63% of total weight) than for the SW-DR methods (31%–33%). The differences in the weights are evidenced in their 95% confidence intervals, which minimally overlap between methods (Table 3). The less important attributes had different weights but were more comparable across methods.

**Figure 3:**
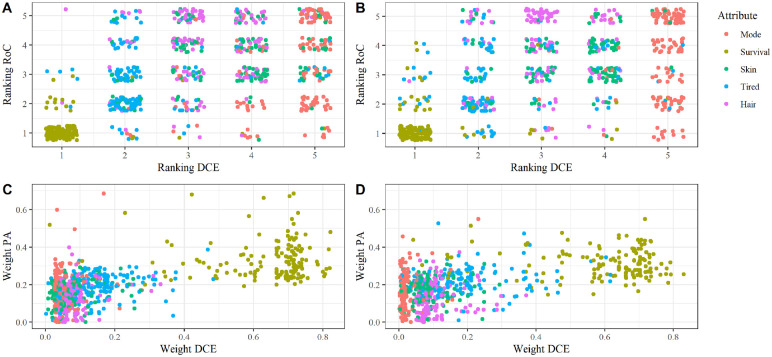
Comparison of the rankings derived from the ROC and DCE in Italy (A) and Belgium (B), and the attribute weighting from the point task and the DCE in Italy (C) and Belgium (D).

The outcomes of the Dirichlet regression models are shown in Table 4. The odds ratio refers to the attribute weights of all attributes relative to 5-y survival of the SW-DR (with the DCE being considered the base case). The aggregate attribute weights of the DCE and SW-DR were significantly different (LL ratio = 466.4 for Italy, *P* < 0.0001; LL ratio = 435.0 for Belgium, *P* < 0.0001). Weights of the SW-DR were more equally divided over the included attributes as compared with the DCE (in the DCE, most of the weight was allocated to the 5-y survival attribute; Figure 3). Relative to survival, the attribute importance weights calculated from the SW-DR for skin problems, mode of administration, tiredness, and hair problems were significantly larger compared wit the DCE weights (*P* < 0.001). Moreover, for Italy, the weights based on the SW-DR were significantly less dispersed (i.e., weighted more equally) compared with the DCE (ϕ = 0.75, CI: 0.64–0.87; *P* < 0.001). These differences remained highly significant even after correcting for educational level, health literacy, gender, age, cancer stage, and treatment experience.

## Discussion

This study used empirical evidence to compare the relative importance of NSCLC treatment profile attribute ranking and weighting when assessed using a DCE or SW-DR task. Significant differences were found in the relative ranking and weights of the attributes between the SW-DR and the DCE. Similar results were found in the 2 countries included in this study, supporting the theoretical validity of these study outcomes. In addition, respondents generally indicated that both DCE and SW-DR tasks were easy or very easy to understand and answer.

The difference in relative attribute weights and ranking is likely in part due to the differences in how the 2 methods assess patient preferences and how respondents engage with the tasks. In an optimally designed DCE, respondents are forced to weigh all attributes when choosing and cannot directly state their individual attribute valuations. In contrast to this multiattribute nature of a DCE, in which the total utility of all attributes guides choices, the SW-DR method is unrestricted, allowing respondents to assign any number of points to attributes (excluding the most important attribute, which automatically receives 100 points).^25^ This may have induced an equivalence bias, leading to a relative undervaluing of the more important attributes and overvaluing of the less important attributes.^53^ The potential presence and impact of equivalence bias in SW experiments should be tested in future research, as the current study was not powered to test conclusions in this regard. Nevertheless, a small explorative post hoc add-on study was conducted (see Appendix B) to explore whether a restricted PA task (forcing respondents to consider all attributes in assigning points) results in more equivalent relative importance weights to the DCE than the unrestricted DR task. In this study, 14 (randomly selected) Italian patients who previously completed the full survey were asked to complete the SW-DR task from the original survey as well as an additional restricted PA task. Respondents were asked to divide a total of 100 points over 5 attributes rather than simply rate each swing on a 100-point scale, thus forcing respondents to account for all attributes when allocating points.^26^ While small and underpowered due to the explorative nature of this study, the results indicate that weights based on this restricted PA task more closely resemble the DCE study outcomes than those from the unrestricted DR task, which replicate previous findings^53^ (see Appendix B). Further studies are needed to confirm if findings from this exploratory analysis hold with larger samples, different sample composition, and different choice contexts to see whether differences remain and compare the outcomes with DCE outcomes.

Surprisingly, respondents did not report the SW-DR method being easier to understand and answer compared with the DCE. While, on one hand, this supports the use of SW-DR in future research on treatment preferences in similar patient populations, it does not favor this method over the DCE. Contrary, one could question whether DCE choice tasks really are as difficult as previously has been assumed. Respondents might be perfectly capable to accurately complete such choice tasks, which would “call for a partial change in perspective toward this method as being (too) complex and time consuming to complete.”^37^ In part, this might be affected by the steep increase in the use of DCEs to elicit preferences,^6^ which has undoubtfully led to increased familiarity among researchers with accurate design and conduct of DCE studies. Given that the SW method is relatively unexplored, this calls for further investigation into how best to design such studies, with specific attention for the validity and reliability of this method in studies aiming to measure attribute relative importance ranking and weights. While awaiting this evidence, the current study outcomes support the use of DCE over SW-DR in preference assessment.

A primary strength of this study is that the empirical evidence used to compare the 2 methods was generated in a 1-time survey of NSCLC patients who completed both methods, allowing for direct comparison of results. The within-subjects design reduced the chance of confounding factors playing a role in different preference outcomes. This survey was developed after an extensive qualitative study in close collaboration with a multidisciplinary team of clinicians, patients, and researchers. The tasks were explained using informational videos designed for the study, and the online setting allowing respondents to pause the educational material or the survey and return to it at a later time in. The online setting also allowed for multicountry, location-independent data collection and access for those with more serious disease complications or fatigue to participate, increasing the generalizability of the findings to other NCSLC populations and reducing the chance of bias.

However, this study also had some limitations. First, SW tasks were originally designed to be conducted in person via a trained facilitator.^26,31^ The current study was administered online, with respondents completing the survey on their own. While online surveys are less costly and time-consuming than interviewer-led studies and SW surveys have previously been done online, the presence of an interviewer allows for assistance and clarification of questions or issues that could arise while the participant is completing the choice task.^69^ This can be especially helpful when attributes are complex or the target population experiences cognitive impairments.^31^ The patient feedback questions indicated that the online setting was not a problem for this study. Second, the sample was composed of relatively old and “fragile” NSCLC patients, reducing generalizability to younger or less fragile patient populations. Generalizability is also limited by the fact that the digital format of the survey may have discouraged those patients with lower digital literacy from participating as well as those who lack access to computer equipment or to the internet.^70^ Third, the current study focused on medical decision making along the medical product life cycle, which did not include clinical or shared decision making. Because other outcome measures and potential methodological considerations might be important when selecting a preference method, the current findings might have limited generalizability toward those situations. Finally, it is unclear whether patients received support from relatives while completing the survey. If this occurred, those supporting the patient in completing the survey could have influenced the outcomes of the survey such that the values measured did not solely reflect the true values of the patient.

In conclusion, this study found significant differences in attribute importance between DCE and SW-DR as well as a greater spread in the DCE-derived relative importance of the attributes. Respondents reported both methods being relatively easy to understand and answer. Further studies confirming these findings as well as SW studies with restricted PA tasks are warranted to enable the provision of accurate guidance for methods selection when studying relative attribute importance across a wide array of preference-relevant decisions. Such studies will contribute to the knowledge base around the validity and reliability of SW in health preference assessment, support guidance for good research practices when using this method, and help researchers decide which method to use when assessing attribute relative importance ranking and weights. While awaiting this evidence, the current study outcomes support the use of DCE over SW-DR in preference assessment.

## Supplemental Material

sj-docx-1-mdm-10.1177_0272989X231222421 – Supplemental material for Comparing Discrete Choice Experiment with Swing Weighting to Estimate Attribute Relative Importance: A Case Study in Lung Cancer Patient Preferences Click here for additional data file. Supplemental material, sj-docx-1-mdm-10.1177_0272989X231222421 for Comparing Discrete Choice Experiment with Swing Weighting to Estimate Attribute Relative Importance: A Case Study in Lung Cancer Patient Preferences by J. Veldwijk, I. P. Smith, S. Oliveri, S. Petrocchi, M. Y. Smith, L. Lanzoni, R. Janssens, I. Huys, G. A. de Wit and C. G. M Groothuis-Oudshoorn in Medical Decision Making

sj-docx-2-mdm-10.1177_0272989X231222421 – Supplemental material for Comparing Discrete Choice Experiment with Swing Weighting to Estimate Attribute Relative Importance: A Case Study in Lung Cancer Patient Preferences Click here for additional data file. Supplemental material, sj-docx-2-mdm-10.1177_0272989X231222421 for Comparing Discrete Choice Experiment with Swing Weighting to Estimate Attribute Relative Importance: A Case Study in Lung Cancer Patient Preferences by J. Veldwijk, I. P. Smith, S. Oliveri, S. Petrocchi, M. Y. Smith, L. Lanzoni, R. Janssens, I. Huys, G. A. de Wit and C. G. M Groothuis-Oudshoorn in Medical Decision Making
