# Supplementary material for: Comparing Discrete Choice Experiment with Swing Weighting to Estimate Attribute Relative Importance: A Case Study in Lung Cancer Patient Preferences
Source: Med Decis Making. 2024 Jan 4;44(2):203–16. doi: 10.1177/0272989X231222421 (PMC10865764; doi:10.1177/0272989X231222421)
Supplement: sj-docx-1-mdm-10.1177_0272989X231222421 – Supplemental material for Comparing Discrete Choice Experiment with Swing Weighting to Estimate Attribute Relative Importance: A Case Study in Lung Cancer Patient Preferences [file sj-docx-1-mdm-10.1177_0272989X231222421.docx]

**Appendix A Model outputs of the DCE and SW analyses**

Table 1. Preference of Italian and Belgian NSCLC patients for treatment profiles, based on DCE

| **Italian data** |  | Mean | | | SD | | |
| --- | --- | --- | --- | --- | --- | --- | --- |
|  |  | *Estimate* | *SE* | *95% CI* | *Estimate* | *SE* | *95% CI* |
| Mode of administration | Oral treatment (ref) | 0 |  |  | 0 |  |  |
|  | Infusion in hospital 12 hours | -0.65*** | 0.16 | -0.97;-0.33 | 0.00 | 0.37 | -0.75;0.76 |
|  | Infusion in hospital 24 hours | -0.64*** | 0.17 | -0.97;0.31 | 0.87*** | 0.20 | 0.47;1.27 |
| 5-Year Survival |  | 0.43*** | 0.05 | 0.33;0.54 | 0.22*** | 0.03 | 0.16;0.28 |
| Risk of long-lasting skin problems |  | -0.04*** | 0.01 | -0.06;-0.03 | 0.04*** | 0.01 | 0.02;0.05 |
| Risk of extreme tiredness |  | -0.06*** | 0.01 | -0.07;-0.04 | 0.04*** | 0.01 | 0.03;0.04 |
| Hair loss | Complete loss of hair (ref) | 0 |  |  | 0 |  |  |
|  | Some hair loss | 0.90*** | 0.16 | 0.58;1.21 | 0.20 | 0.30 | -0.40;0.79 |
|  | No hair loss | 1.41*** | 0.20 | 1.03;1.79 | 0.94*** | 0.24 | 0.48;1.40 |
|  |  |  |  |  |  |  |  |
| Model fit measures: |  |  |  |  |  |  |  |
| Log Likelihood |  | -697.40 |  |  |  |  |  |
| AIC |  | 1422.8 |  |  |  |  |  |
| Pseudo R^2^ |  | 0.47 |  |  |  |  |  |
|  |  |  |  |  |  |  |  |
| **Belgian data** |  | **Mean** | | | **SD** | | |
|  |  | *Estimate* | *SE* | *95% CI* | *Estimate* | *SE* | *95% CI* |
| Mode of administration | Oral treatment (ref) | 0 |  |  | 0 |  |  |
|  | Infusion in hospital 12 hours | -0.09 | 0.18 | -0.43;0.25 | 0.45 | 0.33 | -0.20;1.09 |
|  | Infusion in hospital 24 hours | -0.20 | 0.17 | -0.54;0.14 | 0.55*** | 0.21 | 0.14;0.96 |
| 5-Year Survival |  | 0.39*** | 0.05 | 0.30;0.47 | 0.23*** | 0.04 | 0.15;0.31 |
| Risk of long-lasting skin problems |  | -0.04*** | 0.07 | -0.06;-0.03 | 0.04*** | 0.01 | 0.02;0.05 |
| Risk of extreme tiredness |  | -0.07*** | 0.01 | -0.08;-0.05 | 0.05*** | 0.01 | 0.04;0.07 |
| Hair loss | Complete loss of hair (ref) | 0 |  |  | 0 |  |  |
|  | Some hair loss | 1.16*** | 0.18 | 0.80;1.52 | 0.24 | 0.32 | -0.39;0.86 |
|  | No hair loss | 1.58*** | 0.24 | 1.10;2.05 | 1.12*** | 0.23 | 0.67;1.58 |
|  |  |  |  |  |  |  |  |
| Model fit measures: |  |  |  |  |  |  |  |
| Log Likelihood |  | -698.03 |  |  |  |  |  |
| AIC |  | 1424.1 |  |  |  |  |  |
| Pseudo R^2^ |  | 0.44 |  |  |  |  |  |

***p<0.001

Table 2. Attribute ranks and weights (SD) representing their relative importance based on Swing Weighting (SW) (both Direct Rating (DR) and Rank Ordered Centroid (RoC)) by country.

|  | Italy | | Belgium | | | |
| --- | --- | --- | --- | --- | --- | --- |
|  | RoC | DR | | RoC | DR |  |
| Mode of administration | 3 0.16 (0.12) | 3 0.18 (0.10) | | 3 0.14 (0.12) | 4 0.16 (0.10) |  |
| 5-Year Survival | 1 0.43 (0.08) | 1 0.33 (0.10) | | 1 0.42 (0.10) | 1 0.31 (0.08) |  |
| Risk of long-lasting skin problems | 4 0.14 (0.07) | 4 0.16 (0.06) | | 4 0.14 (0.06) | 3 0.18 (0.06) |  |
| Risk of extreme tiredness | 2 0.18 (0.09) | 2 0.19 (0.07) | | 2 0.20 (0.08) | 2 0.22 (0.07) |  |
| Hair loss | 5 0.10 (0.08) | 5 0.14 (0.07) | | 5 0.11 (0.10) | 5 0.13 (0.08) |  |
