# Supplementary material for: Comparing Discrete Choice Experiment with Swing Weighting to Estimate Attribute Relative Importance: A Case Study in Lung Cancer Patient Preferences
Source: Med Decis Making. 2024 Jan 4;44(2):203–16. doi: 10.1177/0272989X231222421 (PMC10865764; doi:10.1177/0272989X231222421)
Supplement: sj-docx-2-mdm-10.1177_0272989X231222421 – Supplemental material for Comparing Discrete Choice Experiment with Swing Weighting to Estimate Attribute Relative Importance: A Case Study in Lung Cancer Patient Preferences [file sj-docx-2-mdm-10.1177_0272989X231222421.docx]

**Appendix B. Description and results of add-on study**

A random sample of 14 respondents who initially participated in the study in Italy were recontacted. Upon providing informed consent, respondents were asked to read the instruction materials (including the educational videos) for the SW again. Respondents completed the SW and DR tasks as also included in the original survey. After that, new SW instructions were incorporated explaining the revised ranking exercise (point allocation (PA). Respondents were asked to complete this PA task by dividing 100 points over the five attributes reflecting their importance. Data of the SW-DR and SW-PA were analyzed according to the analyses described in the main paper. Outcomes of the SW-DR and SW-PA were compared against each other as well as compared against the DCE outcomes of the original study.

Table 1 shows the outcomes of the SW-DR, the SW-PA and the DCE outcomes. On average we can see that the SW-PA weights for the attributes differ from those of the SW-DR and are more in resemblance with the weights obtained from the DCE.

Table B1. Relative attributes weights from the SW-DR, SW-PA of add-on study and DCE of original study

|  | SW-DR | SW-PA | DCE |
| --- | --- | --- | --- |
| Mode of administration | 0.12 | 0.06 | 0.04 |
| 5-Year Survival | 0.37 | 0.77 | 0.65 |
| Risk of long-lasting skin problems | 0.16 | 0.06 | 0.08 |
| Risk of extreme tiredness | 0.22 | 0.07 | 0.16 |
| Hair loss | 0.13 | 0.04 | 0.07 |
